# Unusual phosphaturic mesenchymal tumor mimicking osteoid osteoma

**DOI:** 10.1016/j.radcr.2023.05.008

**Published:** 2023-06-03

**Authors:** Elsa Hervier, Karel Gorican, Sana Boudabbous, Emmanuel Biver, Serge Ferrari, Essia Saiji, Valentina Garibotto, Ismini Mainta

**Affiliations:** a Division of Nuclear Medicine, Diagnostic Department, Geneva University Medical Center, Faculty of Medicine, Geneva University Hospitals, University of Geneva, Rue Gabrielle-Perret-Gentil 4, CH-1211 Geneva, Switzerland; b Division of Radiology, Diagnostic Department, Geneva University Hospitals, Geneva, Switzerland; c Faculty of Medicine, University of Geneva, Geneva, Switzerland; d Division of Bone Diseases, Geneva University Hospitals, Geneva, Switzerland; e Division of Clinical Pathology, Diagnostic Department, Geneva University Hospitals, Geneva, Switzerland

**Keywords:** Phosphaturic mesenchymal tumor, Fibroblast growth factor 23, Tumor-induced osteomalacia, Ga-68 DOTATATE PET-CT, MRI, Bone tumors

## Abstract

Phosphaturic mesenchymal tumor is a rare tumor characterized by paraneoplastic osteomalacia. The diagnosis is often delayed because of nonspecific symptoms and difficulty to localize the tumor. In this study we report a case of PMT of the left femur detected by Ga-68-DOTATATE PET-CT with radiological features mimicking osteoid osteoma. We report a 31-year-old female patient who presented to our hospital for evaluation due to progressive bone pain and muscle weakness. Her laboratory data showed hypophosphatemia and increased fibroblast growth factor 23 (FGF-23) together with reduced bone mineral density on bone densitometry. The diagnosis of PMT was suspected and the tumor was identified on Ga-68-DOTATATE PET-CT as a focal uptake in a lucent lesion of the left femoral head with a central sclerotic dot mimicking a nidus as seen in osteoid osteoma. The lesion was treated with percutaneous radiofrequency ablation. Laboratory tests and bone densitometry rapidly improved post-treatment. The present case emphasizes the difficulty to diagnose PMT due to its nonspecific biochemical and clinical presentation and the relevance of functional imaging for locating these tumors despite different radiological presentation.

## Introduction

Tumor-induced osteomalacia (TIO) also known as oncogenic osteomalacia is a rare paraneoplastic syndrome due to phosphaturic mesenchymal tumor (PMT). Wiedner and Santa Cruz coined the term PMT in 1987. Most tumors occur in middle-aged adults and are more prevalent in males, but it has also been described in the pediatric and elderly population. Less than 1000 cases have been reported in the literature, thus the incidence has not been established yet. These tumors arise from mesenchymal tissue and are mainly benign with less than 10% of all reported cases being malignant [1].

We present a patient with a rare bone PMT detected on Ga-68-DOTATATE PET-CT demonstrating radiological features of an osteoid osteoma (OO). This case emphasizes that Ga-68-DOTATATE PET is the imaging of choice and highlights the input of percutaneous treatment with good clinical and biological outcome.

## Case report

We present a 31-year-old woman with no medical history who suffered from painful nonunion traumatic rib fractures after a car accident 7 years earlier, chronic lower back pain, left hip pain, and muscle weakness over the past 5 years, and more recently a delayed osseointegration after dental implants.

In this context, the patient underwent a bone densitometry which showed reduced bone density with a T-score of −3.0 standard deviations (SD) at the femoral neck and −2.3 SD at the lumbar spine. The baseline laboratory revealed hypocalcemia (2.02 mmol/L; reference range 2.20-2.52 mmol/L), decreased calcitriol (31 nmol/L; reference range 48-168 pmol/L) and 25-hydroxy vitamin D (29 nmol/L; reference range >75 nmol/L), elevated serum alkaline phosphatase (ALP) (280 IU/L; normal range 30-140 IU/L), and elevated beta-crosslaps (1.10 µg/L; reference range <0.57 µg/L), while parathormone (PTH) was in the normal range (62.1 ng/L; reference range 18.4-80.1 ng/L), with preserved kidney function (eGFR 121 mL/min/1.72 m^2^; reference range >60).

**Fig. 1:**
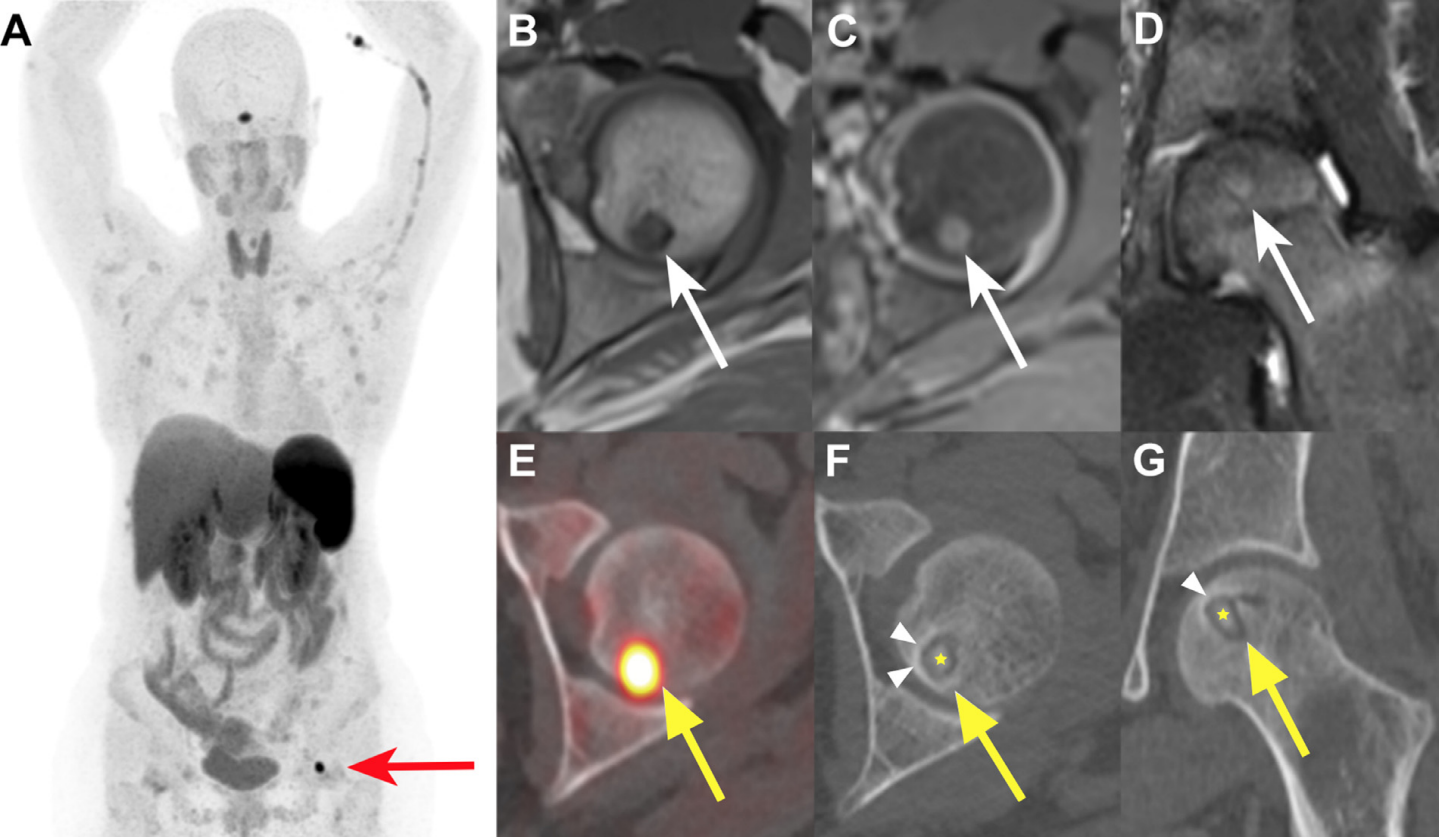
(A) Maximum intensity projection (MIP) of Ga-68-DOTATATE PET images 60 minutes after radiotracer injection showing a well-defined focus of markedly increased radiotracer uptake on the left femoral head (*red arrow*); (B and C) axial MRI images of pre- and postcontrast T1-weighted sequences revealing an 8 mm eccentric epiphyseal lesion of the left femoral head, adjacent to the articular surface, hypointense with a thin sclerotic rim, a peripheral contrast enhancement and (D) on coronal MRI images of STIR sequence demonstrating a slight hyperintensity (*white arrows*); (E) axial Ga-68-DOTATATE PET/CT fusion image showing the focus of increased radiotracer uptake on the left femoral head lesion with standardized uptake value reaching a maximum of 25.6 (*yellow arrow*); (F and G) axial and coronal CT images illustrating the culprit tumor on the left femoral head (*yellow arrow*) as a well circumscribed lucent lesion surrounded by a sclerotic rim (*white arrow heads*) and a central sclerotic dot mimicking a nidus (*yellow star*).

Osteomalacia resulting from nutritional deficiencies was suspected and the patient was prescribed with oral vitamin D and calcium supplementation with progressive dose-escalation until a maximum of calcium 2000 mg and cholecalciferol 2800 UI. To further explore the symptoms, a spine MRI was performed which did not reveal pathological fractures nor signs of spondylarthropathy but the presence of a rugger jersey appearance of the spine often related to hyperparathyroidism. A pelvic MRI showed a small eccentric epiphyseal lesion of the left femoral head, adjacent to the articular surface, measuring 8 mm, markedly hypointense with a thin sclerotic rim on T1-weighted sequence, slightly hyperintense on T2-weighted sequence, and a peripheral contrast enhancement, interpreted as a geode or subchondral cyst (Fig. 1).

**Fig. 2:**
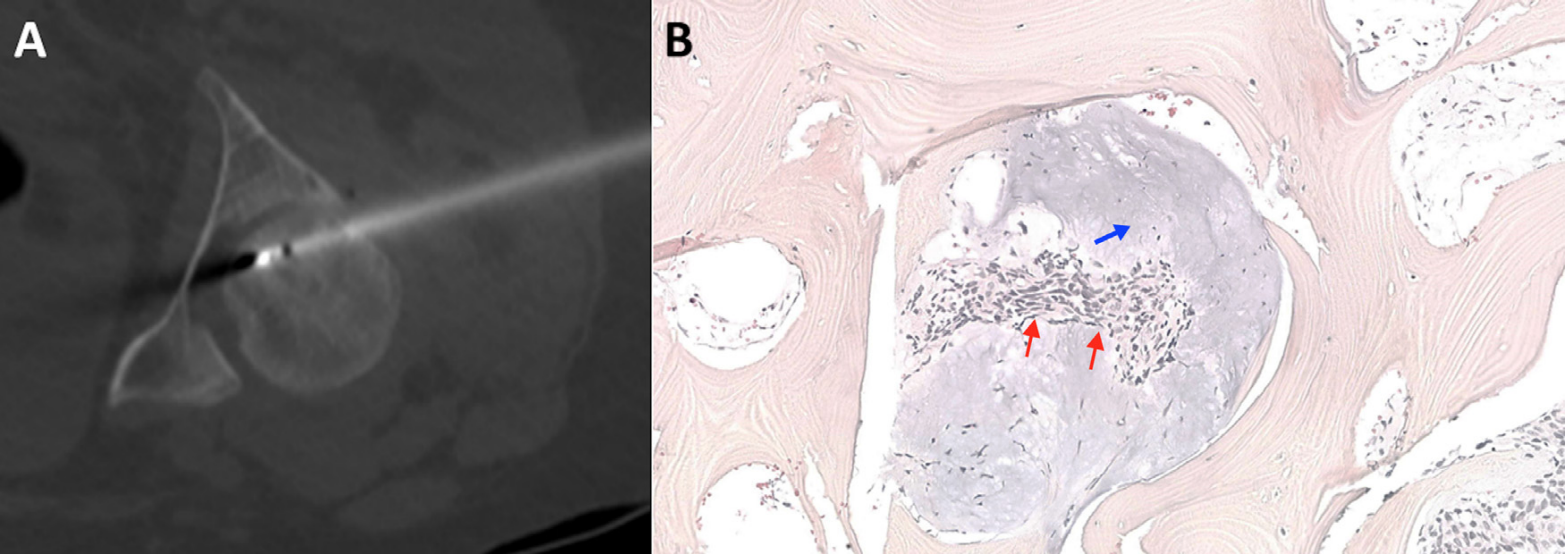
(A) Axial view of the CT during RFA showing the needle in the center of the lesion. (B) High power view showing bland spindled cells (*red arrows*) and basophilic myxoid matrix (*blue arrow*), filling the osteomedullary spaces.

Despite a well-conducted treatment, the patient's symptomatology worsened and the biochemical parameters did not improve, with the onset of transitory hyperparathyroidism and severe persistent hypophosphatemia not measured previously (0.59 mmol/L; reference range 0.80-1.45 mmol/L). As the patient also presented iron deficiency, malabsorption was explored and excluded with endoscopic biopsies and MRI. Renal phosphate losses were then evaluated, with the tubular maximum reabsorption of phosphate showing a decreased value of 0.36 mmol/L (normal range 0.8-1.35 mmol/L) despite normalization of calcitriol (83 pmol/L; reference range 48-168 pmol/L). In this context, an estimation of the plasma c-terminal fibroblast growth factor 23 (FGF-23) was performed, revealing its abnormal increase (66.1 pg/mL; reference range 10-50 pg/mL), thus suggestive of an FGF-23-related hypophosphatemia inducing osteomalacia presumably secondary to a PMT. To confirm this suspicion, a functional imaging with Ga-68-DOTATATE PET-CT was performed as it is known to have an excellent sensitivity and specificity for these tumors. This exam revealed a focus of increased uptake on the left femoral head, corresponding to the lesion previously seen on MRI, presenting on CT as a well circumscribed lucent lesion surrounded by a sclerotic rim and a central sclerotic dot mimicking a nidus as seen in osteoid osteoma (Fig. 1). A bone biopsy was performed, which showed bland stellate to short spindle cells, filling the medullary spaces, set in a background of myxoid to hyalinized matrix (Fig. 2). The cells showed immunohistochemical expression of SATB2. The RNA fusion panel found a *FOSB*::*MYADM* transcript. The histologic features could reasonably exclude the diagnosis of osteoid osteoma.

Despite the nonspecific molecular finding, the diagnosis of PMT was retained and a Ga-68-DOTATATE PET-CT was repeated 2 months later, showing a persistent single increased focal uptake on the left femoral head lesion, unchanged, still suspicious for a PMT with TIO.

After discussion with the orthopedic team, surgical treatment with total hip replacement was dismissed because of the patient's young age and fragile bone, and she underwent CT-guided radiofrequency ablation instead (RFA).

**Fig. 3:**
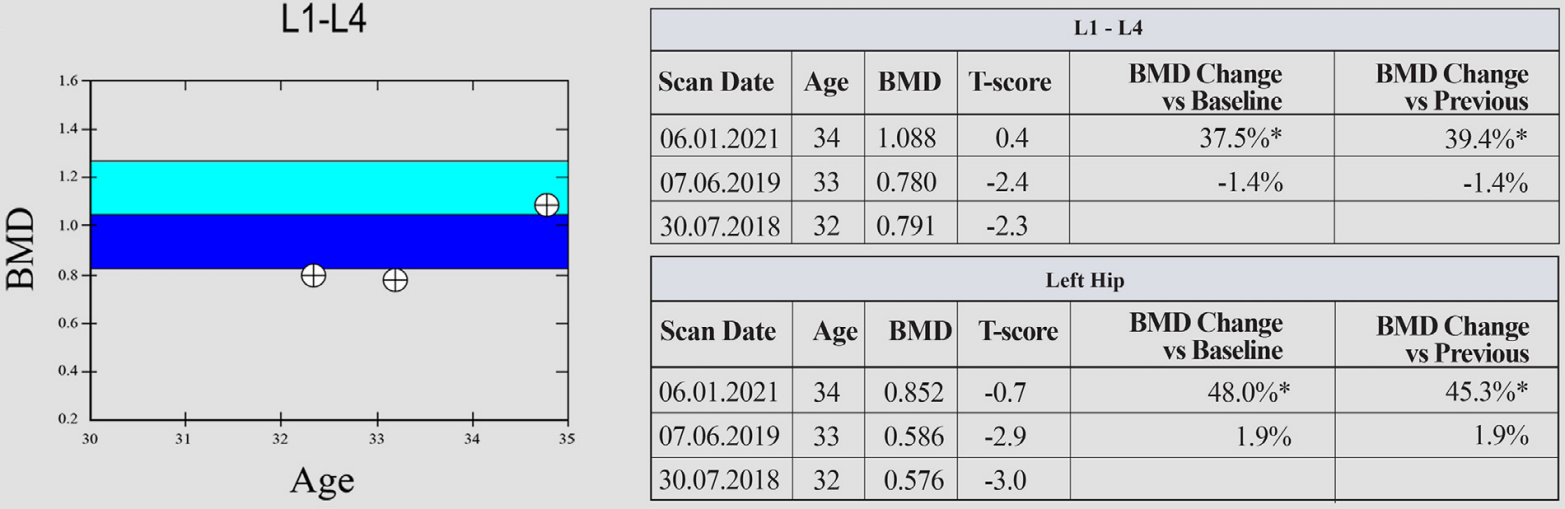
Bone densitometry results demonstrating a significant increase of T-score both on the hip and lumbar spine 6 months after radiofrequency ablation of the lesion.

Following this procedure, laboratory showed a normalization of phosphatemia (1.43 mmol/L) (Table 1) and the bone density improved of about 45% on T-score in the next 6 months (Fig. 3). During the 3 years follow-up, the patient did not show any signs of recurrence.

**Table 1:** Laboratory findings.

Laboratory		Normal range	Pre-treatment	Post-treatment
Blood	Total calcium	2.1-2.6 mmol/L	**2.03**	2.24
	Corrected calcium	2.1-2.6 mmol/L	**2.05**	2.18
	Phosphates	0.87-1.45 mmol/L	**0.62**	1.43
	Calcitriol (1,25-di-OH-vit D)	48-168 pmol/L	83	61
	Parathormone	18.4-80.1 ng/L	30.2	28.5
Urinary	Tubular maximum reabsorption of phosphate	0.8-1.35 mmol/L	**0.36**	NA

## Discussion

PMTs are a rare mesenchymal tumor frequently associated with TIO due to the excessive secretion of FGF-23, a substance recognized as the principal regulator of phosphate homeostasis. FGF-23 binds to FGFR1 receptor, decreases renal phosphate resorption in the proximal tubule, and inhibits 1-alpha-hydroxylase thus reducing calcitriol (active form of vitamin D) [2]. Since calcitriol promotes intestinal phosphate absorption, patients with PMT also have difficulties absorbing phosphate from their diet. In turn, those pathophysiological mechanisms will result in increased phosphate excretion, mobilization of calcium and phosphate from bones, decreased osteoblastic activity, and finally osteomalacia in adults and rickets in children [3].

Classically blood laboratory show low or inappropriately low-normal calcitriol, hypophosphatemia, elevated bone fraction of ALP, normal calcium and PTH levels, together with elevated levels of urine phosphate. In some cases, PTH can be elevated, reflecting secondary hyperparathyroidism caused by low levels of calcitriol [4] and occasionally patients can develop tertiary hyperparathyroidism especially when they receive phosphorus supplementation without activated vitamin D for a long period [5]. Finally a positive correlation has been observed between FGF-23 levels and tumor size [6].

The diagnosis is often delayed because it is a rare disease with unspecific symptoms including severe bone and muscle pain, fatigue, and pathological fractures. The average time to the correct diagnosis after symptoms onset is 2.9 ± 2.3 years [7]. The detection of these tumors is challenging because of their small size, slow-growing nature, and atypical location. PMTs are slightly more often found in bone than soft tissue and frequently arise in the extremities and craniofacial bones [8]. Thorough head-to-toe physical examination can sometimes localize the tumor and if no masses are identified an imaging approach can be undertaken [4].

Functional imaging is used to further explore this pathology primarily with Ga-68 DOTATATE, a somatostatin analog with high affinity for the somatostatin receptor 2, overexpressed in PMTs [9]. Once bound to the receptor, the complex formed with the radiotracer is internalized resulting in high accumulation within tumor cells. In comparison, the detectability of PMT through glucose metabolism with 18F-FDG is only limited due to its low proliferative rate resulting in modest level of uptake. In a recent meta-analysis, the pooled sensitivity of Ga-68 DOTA-SSTR PET-CT for the detection of PTM was 86% (95%CI: 79%-91%) with a pooled specificity of 86% (95%CI: 57%-98%), compared to 73% (95%CI: 61%-84%) sensitivity and 36.3% specificity for F-18-FDG PET-CT [6]. Another meta-analysis including also Octreoscan SPECT-CT (a somatostatin analog labeled with Indium-111, a gamma-ray-emitting isotope) found 90% (95%CI: 82%-95%) sensitivity for Ga-68 DOTA-SSTR PET-CT, 83% (95%CI: 75%-89%) for Octreoscan SPECT-CT, both significantly superior than F-18-FDG PET-CT (67%, 95%CI: 53%-80%; *P* < .005). Although, the difference in sensitivity between Ga-68 DOTA-SSTR PET-CT and Octreoscan was not significant (*P* = .161) [10], Ga-DOTA-SSTR PET-CT has higher affinity for SSTR, better spatial resolution and more rapid total body imaging, making functional imaging with Ga-68 DOTA-SSTR PET the modality of choice for the detection of culprit tumors [11], [12], [13]. As a result, whole body Ga-68-DOTATATE PET-CT is recommended as a first-line imaging, but this indication remains off-license. When the tumor is not identified and symptoms persist, it is advised to repeat imaging every 1-2 years as some tumors will eventually grow and be located [3].

Once the culprit tumor has been identified, anatomic imaging is used to characterize the lesion, including CT and MRI. When compared with OO which typically presents on CT as a lucent lesion with a central nidus mineralization and surrounding sclerotic reactive bone [14,15], the radiographic osseous pattern of bone PMT is generally nonspecific. It mostly manifests as an osteolytic lesion and less commonly osteosclerotic or mixed lesion with a narrow transition zone. Some PMTs might also have ground glass appearance and can be confused with fibrous dysplasia [16]. On MRI, PMTs and OO present similar characteristics, generally isointense to muscle on T1-weighted imaging and markedly hyperintense on T2-weighted imaging [17]. On postinjection T1-sequences most PMTs and OO show uniform enhancement, with a partial venous washout in OO [16,18]. One notable difference is that OO can present with a surrounding bone marrow edema detected on T2-weighted imaging and postinjection T1-weighted sequences, and it can also present with a target-like appearance as the mineralized central portion shows low signal intensity on all sequences and does not enhance [19].

Histologically, PMTs can have a heterogeneous morphology with variable patterns, but usually show bland, spindle to stellate cells, which produce a characteristic hyalinized to smudgy-appearing matrix. Detection of *FN1*::*FGFR1* or *FN1*::*FGF1* fusion is not essential for the diagnosis, as it has been reported in only 42% and 6% of PMT cases, respectively [20]. Clinical evidence of osteomalacia, corrected by complete excision of the tumor, remains an important criterion to be associated with histological features, in order to make the diagnosis. The detection of novel fusions may be useful for the routine diagnosis of PMT lacking *FN1*::*FGFR1*/*FGF1* fusion.

As a differential diagnosis, TIO with elevated FGF-23 levels has also been described with different types of carcinomas including colonic adenocarcinoma, ovarian serous carcinoma, clear cell renal carcinoma, lung small cell carcinoma, and anaplastic thyroid carcinoma [21], [22], [23], [24], [25]. To our knowledge no FGF-23-mediated TIO-like paraneoplastic syndrome has been reported with osteoid osteoma.

Complete surgical resection with wide margins is the current standard of care and is the only definitive therapy of TIO [4,^8^,26]. In case of subtotal resection, there have been reports of persistent symptoms, local recurrence or distant metastasis even in benign lesions [27,28]. Alternative treatments include definitive and adjuvant radiotherapy [29], [30], [31], cryoablation and destruction by radiofrequency ablation as for other benign musculoskeletal tumors such as osteoid osteoma [17,[32], [33], [34]. Percutaneous treatment is less invasive and the risk of iatrogenic complication such as fracture is reduced. This procedure is often CT-guided and performed on outpatient basis.

Finally, when the tumor cannot be identified nor resected, apart from the classical medical treatment with phosphate and activated vitamin D, new therapies have emerged especially Burosumab, a fully human monoclonal antibody against FGF-23, first approved for the treatment of X-linked hypophosphatemia in children and adults, which has demonstrated a good improvement of biochemical and clinical symptoms in TIO [35,36].

After treatment, serum FGF-23 level falls rapidly and phosphate returns to normal during the first week postsurgery and patient's symptoms regress within days to weeks after tumor removal as in our case. Follow-up of those patients should be done as delayed metastasis has been observed [37].

## Conclusion

PMT is a rare tumor but should be considered into the differential diagnosis of osteomalacia with hypophosphatemia. Whole-body Ga-68-DOTATATE PET is recommended as the first-line imaging for these tumors. Treatment with RFA is an effective alternative to surgery when it cannot be performed.

## Authors’ contributions

All listed authors have substantially contributed in the conception and the creation of the article and have approved the final version of the manuscript. The participant has given written informed consent for publication.

## Patient consent

Written informed consent was obtained from the patient(s) for publication of this case report, including accompanying images.
